# Giant Coronary Artery Aneurysm Due to Implantation of Drug-Eluting Stent

**DOI:** 10.7759/cureus.52004

**Published:** 2024-01-10

**Authors:** Ahmad Amir, Abbas Ali Qayyum

**Affiliations:** 1 Cardiology, Rigshospitalet, Copengagen, DNK; 2 Cardiology, Hvidovre Hospital, Copenhagen, DNK

**Keywords:** cypher stent, ischemic heart diseas, stent aneurysms, sirolimus-eluting stent, coronary artery aneurysm, giant coronary artery aneurysm

## Abstract

Development of coronary artery aneurysm after implantation of a drug-eluting stent is a rare complication. The mechanism behind aneurysm formation is unknown, but studies suggest hypersensitivity and inflammatory reactions elicited by the stent polymer. Here, we report a case of a 57-year-old man who was treated with a sirolimus-eluting Cypher^TM^ stent in the left anterior descending artery due to stable angina pectoris and left circumflex artery due to dissection. Coronary aneurysm formation at the site of stent implantation was discovered three years after the stents were deployed, and progression of the aneurysms was seen in the coronary artery angiography. We hypothesize about the mechanism of aneurysm formation and present management of the aneurysms.

## Introduction

Drug-eluting stents (DES) used for percutaneous coronary intervention (PCI) comprise the supporting structure of a metal stent, a polymer that is the drug carrier vehicle, and an anti-restenotic drug embedded within the polymer. All commercially available DES are based on the same general components but differ in the alloy platform, type of polymer, and the anti-restenotic drug used [1].

The sirolimus-eluting Cypher^TM^ stent (Cordis Corp) is a first-generation DES approved by the Food and Drug Administration (FDA) in April 2003. The platform is made of stainless steel with a strut thickness of 140μm. The durable polymer is a mixture of polyethylene-co-vinyl acetate (PEVA) and poly-n-butyl methacrylate (PBMA). Sirolimus is embedded in the polymer and has a drug elution time of 90 days [2]. 

A coronary artery aneurysm (CAA) is defined as an abnormal dilatation of a coronary artery segment exceeding 50% of the adjacent normal segment diameter. If the CCA diameter exceeds the normal segment diameter by greater than 4 times or the diameter is greater than 20 mm, it can be termed a giant CAA (GCAA), but there is no clear consensus on the definition [3].

The overall incidence of CAA ranges from 0.3% to 5.3%, and GCAA is far less common, with an incidence of 0.02%. CAA is a rare complication after DES implantation, with reported incidence ranging from 1.25% to 3.9% [4]. Risk factors for CAA development after DES implantation are lesion length > 33 mm, lesion on the left anterior descending artery (LAD), chronic total occlusion, and implantation in an infarct-related artery [5].

The mechanism for developing CAA after DES implantation is not well understood, but several hypotheses have been proposed. Residual dissection and deep arterial wall injury during high-pressure balloon inflations and stent implantation have been proposed to explain aneurysm formation. In addition to the mechanical risk factors, an altered vessel wall environment at the site of the implanted DES is thought to cause CAA by delayed reendothelialization and neointimal healing, inflammatory changes of the medial wall, and hypersensitivity reactions to the drug or polymer mixture [4,6-7]. In particular, the polymer has been shown to induce a marked inflammatory response, resulting in increased eosinophilic/heterophilic infiltration into the vessel wall. This is supported by several animal studies and human autopsy studies [6-9]. These changes in the arterial wall environment are proposed to cause disruption and weakening of the arterial wall, leading to expansion and predisposing to aneurysm formation [4,7].

Here, we report a case of two CCA's developed at the site of Cypher^TM^ stent implantation, which were managed with anticoagulant and antiplatelet therapy.

## Case presentation

A 47-year-old man with a medical history of hypertension and dyslipidemia was first admitted to the hospital in March 2010 due to stable angina pectoris. The patient was a heavy smoker with mild obesity (Body Mass Index 32.5) without a history of diabetes or heart disease. Diagnostic coronary angiography (CAG) revealed significant stenosis in the proximal LAD with a fractional flow reserve (FFR) of 0.71 and a 50% stenosis in the proximal left circumflex artery (LCx) with an FFR of 0.88. PCI was successfully performed with a 3.5 mm x 28mm Cypher stent implantation in proximal LAD. The procedure was uncomplicated, and the patient was discharged on dual antiplatelet therapy (aspirin and clopidogrel) and atorvastatin for regular follow-up.

**Figure 1:**
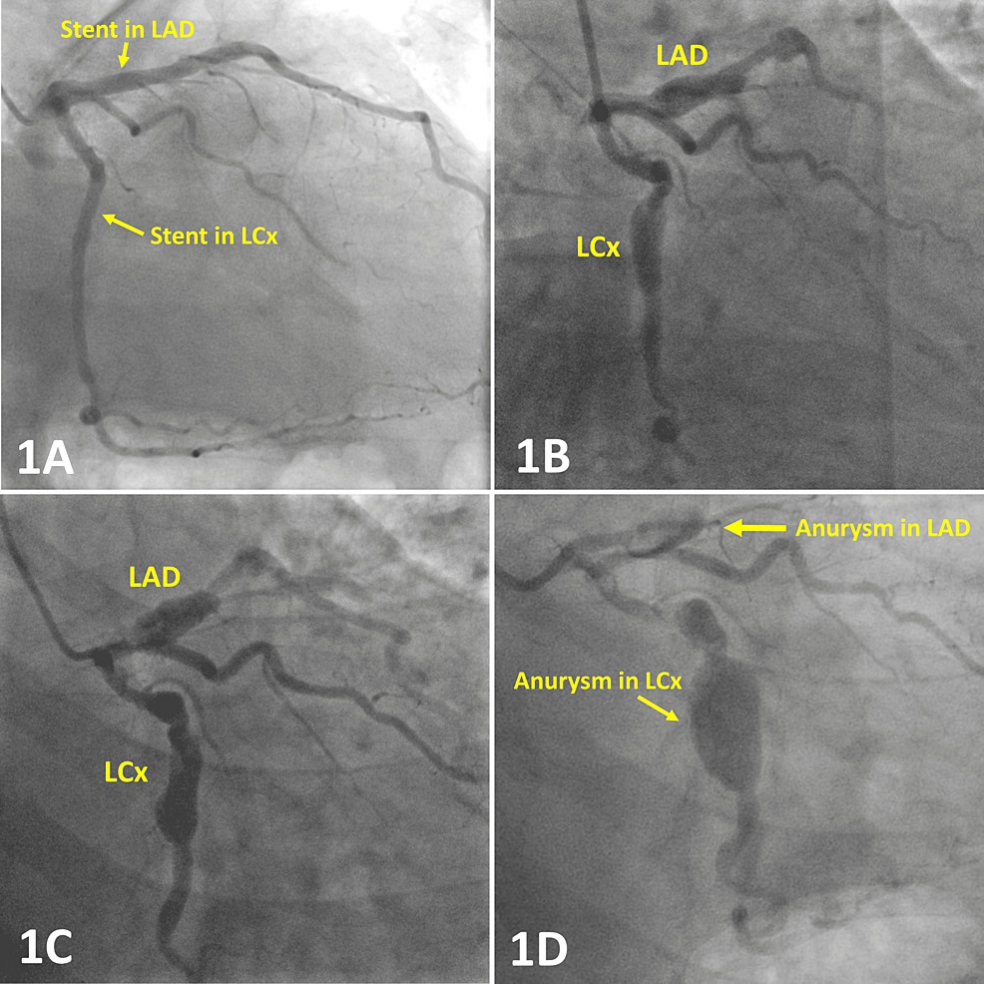
Coronary angiography showing aneurysm formation in LAD and LCx Figure 1A. CAG from October 2010 showed no sign of aneurysm formation in relation to Cypher stents in proximal LAD and LCx. Figure 1B. CAG from 2013 revealed aneurysm formation in relation to Cypher stents in proximal LCx and LAD. Figure 1C. CAG from 2017 shows the progression of both aneurysms in LCx and LAD, Figure 1D. CAG from 2021 shows further progression of both aneurysms, especially the aneurism in LCx, CAG: coronary angiography; LAD: left anterior descending artery; LCx: left circumflex artery

Two months later (may 2010), the patient was hospitalized with non-ST-elevation myocardial infarction (nSTEMI), and CAG revealed significant bifurcation stenosis in LCx and the first marginal artery (M1). PCI was performed using the crush technique with stents from Taxus and Endeavor. The procedure was complicated with dissection in the middle LCx, distal to the implanted stent, which was treated with Cypher 3.0 mm x 33 mm stent with a good visual result. A follow-up CAG in October 2010 showed well-deployed Cypher stents in proximal LAD and mid-LCx, with no residual stenosis (Figure 1A).

In 2013, the patient was again hospitalized with stable angina pectoris, and PCI was successfully performed with the implantation of a Nobori stent in the middle-distal right coronary artery (RCA). The CAG revealed aneurysm formation at the site of the Cypher stents in LAD and LCx (Figure 1B). The dual antiplatelet therapy with Aspirin and Clopidogrel was converted to lifelong therapy.

In 2017, the patient was hospitalized under suspicion of cardiac syncope. CAG revealed stenosis in the distal part of RCA, and PCI was performed with the implantation of two Biomatrix stents. In addition, the CAG revealed severe aneurysms about the Cypher stents in both LAD and LCx (Figure 1C). The procedures and the stents implanted are summarized in Table 1.

**Table 1:** Summarizes the performed procedures, stent types, and anticoagulant therapy AFib: Atrial fibrillation; Ant STEMI: anterior ST-elevation myocardial infarction; CAG: coronary angiography; LAD: left anterior descending artery; LCx: left circumflex coronary artery; M1: first marginal artery; SAP: stable angina pectoris; POBA: plain old balloon angioplasty; UAP: unstable angina pectoris

Date of procedure	Indication	Coronary artery intervention	Stent type and size	Anticoagulant therapy
March 2010	SAP	Proximal LAD	Cypher (3.5 x 28 mm)	Aspirin and Clopidogrel
May 2010	SAP	LCx-M1 bifurcation and proximal LCx	Taxus (2.25 x 8 mm), Endeavor (3.5 x 12 mm) and Cypher (3.0 x 33 mm)	Aspirin and Clopidogrel
June 2010	Dyspnea	CAG without intervention	-	Aspirin and Clopidogrel
October 2010	UAP	POBA of proximal LCx and proximal OM1	-	Aspirin and Clopidogrel
July 2013	UAP	Middle-distal RCA	Nobori (3.0 x 28 mm), Nobori (3.0 x 14 mm) and Nobori (3.0 x 11 mm)	Aspirin and Clopidogrel
June 2017	Syncope	Distal RCA	Biomatrix (3.5 x 18 mm) and Biomatrix (4.0 x 18 mm)	Aspirin and Clopidogrel
January 2021	Ant STEMI	LAD	POBA	Apixaban and Clopidogrel

In the meantime, the patient was diagnosed with chronic obstructive pulmonary disease (COPD) and type 2 diabetes. In 2019, the patient was diagnosed with atrial fibrillation and dual antiplatelet therapy was converted to single therapy with Apixaban 5 mg x 2. The same year, the patient developed a minor stroke and achieved full recovery after rehabilitation. There was no change in anticoagulant therapy with Apixaban.

In January 2021, the patient was brought to the hospital with anterior ST-elevation myocardial infarction (STEMI). CAG revealed stent thrombosis in LAD with TIMI Coronary Grade Flow 0 (represents total occlusion) and severe aneurysms in relation to Cypher stents. Occlusion in LAD was successfully treated with plain old balloon angioplasty (POBA). LAD was unsuitable for stent implantation due to the extensive aneurysm formation (Figure 1D). After three days of observation, the patient was discharged from the hospital for further follow-up.

The heart team evaluated the patient and determined that the aneurysms' size and morphology were unsuitable for PCI. As the patient didn’t exhibit symptoms related to the aneurysms, surgical treatment was not considered at that time, but it remains an option in case of further progression of the aneurysms. Instead, the heart team decided on conservative management with regular follow-up and lifelong dual antiplatelet therapy. Due to atrial fibrillation, Apixaban and Clopidogrel were administered. The patient is doing well on follow-up after two years, with no recurrence of symptoms.

## Discussion

Although aneurysm formation is a rare complication after PCI with drug-eluting stents, a growing number of case reports document the formation of CAA after DES implantation [5,10].

CAA is defined as focal dilatation of a coronary artery segment >1.5-fold the normal size of adjacent segments. Depending on the integrity of the coronary artery wall, they can be classified as true or false aneurysms (pseudoaneurysms). CAAs are then divided into saccular aneurysms if the longitudinal diameter is less than the transverse diameter and fusiform aneurysms in the opposite condition. However, there is no data regarding the prognostic importance of the aneurysm shape [3]. Both aneurysms in the present case have a fusiform morphology. The aneurysm in LCx measures 22 mm in diameter and may be considered a GCAA. A limitation of this report is that intravascular ultrasound (IVUS) was not performed. IVUS would have provided better characteristics of the aneurysms from our case.

Most CAAs are asymptomatic, and the CAA may be discovered incidentally during angiography, but some patients present with angina pectoris, dyspnea, arrhythmias, myocardial infarction, and congestive heart failure. GCAA are more often symptomatic [3]. In this case report, the CAAs were detected during angiography performed due to stable angina pectoris. To the best of our knowledge, the patient didn't present symptoms due to the aneurysms.

During the seven years from 2010 to 2017, the patient was treated with a total of nine stents from different manufacturers (Taxus, Endeavor, Cypher, Nobori and Biomatrix). Both Cypher stents were implanted in 2010, and there were no signs of aneurysm formation before the stent implantation. CAG performed in 2013 revealed two significant aneurysms in relation to the Cypher stents in LCx and LAD. In 2017 and 2021, CAG revealed progressive growth of both aneurysms, most significantly in LCx, which had grown massively and measured 12 x 22 mm. Interestingly, no aneurysm formation has been detected concerning the other stents our patient had received. This could be due to a vascular response specific to the Cypher stents implanted.

The Cypher stent elutes the sirolimus gradually at the local site over a few months, whereas the stent and polymer persist in the vascular wall [3]. In our case, CAG seven months after the implantation of the first Cypher stent in LAD, and thereby long after total elution of sirolimus, didn't reveal aneurysm formation (see Figure 1A).

Several histological studies have shown marked inflammation surrounding the polymer after DES implantation [6-9]. A case of progressive aneurysm formation and late stent thrombosis in relation to Cypher stents in LCx resulted in fatal myocardial infarction. The following autopsy showed extensive inflammation surrounding the polymer fragments in the Cypher stent, suggesting the stent polymer was the causative factor [9]. In our case, a vascular inflammatory response to the polymer is a plausible cause for the emergence of the aneurysms detected.

Currently, there is no consensus on the optimal treatment of CAA due to a lack of randomized trials or large-scale data, and most of the current recommendations are based on small case series. Treatment options consist of surgical, percutaneous, and medical management. The treatment strategy is individualized for each patient, considering aneurysm size, location, morphology, and expansion over time. The decisions around treatment are also based on the the patient's characteristics, comorbidities, severity of coexisting atherosclerosis, and symptomatic features [3-4,10]. In our case, the heart team explored all the therapeutic options.

Long-term antithrombotic therapy, usually dual antiplatelet therapy, is recommended in patients with CAA after DES implantation. Statins are proposed to have a therapeutic place due to the inhibition of metalloproteinases possibly involved in the inflammatory reaction in CCA. In addition, statins are also recommended because atherosclerosis is associated with CAA formation [3,4]. Angiotensin-converting enzyme inhibitors are also suggested to prevent or slow the progression of CAA, but this has not been proven in long-term studies [10].

Intervention is considered in patients with certain high-risk clinical and/or anatomical features. Percutaneous exclusion with covered stents or coil embolization is conventionally preferred to exclude smaller CAA in patients with suitable anatomy. Surgical management is generally accepted as the preferred treatment for GCAA at high risk of rupture, CAA involving the left main coronary artery, multiple CCA's, and symptomatic patients that cannot be treated percutaneously. Surgical intervention may include aneurysm resection, ligation or marsupialization, and aneurysmectomy with or without bypass grafting [3-4,10]. In our case, the heart team decided on medical therapy, but surgical treatment will be considered in case of symptoms or further progression of the aneurysms.

Some aneurysms resolve naturally or remain stable, but aneurysms can, in rare cases, lead to life-threatening complications such as dissection, rupture, thrombosis, and even sudden death [3,6]. Studies have shown conflicting results on the prognostic impact of CAA after percutaneous coronary intervention. However, recent long-term studies found increased mortality and major adverse cardiac events [10].

## Conclusions

CAA formation after DES implantation is a rare complication but may be underdiagnosed due to its commonly asymptomatic nature. In this case, we present a patient with delayed CAA development at the sites of Cypher stent in LCx and LAD. The exact mechanism responsible for CCA formation after DES remains unknown, but the stent polymer may be involved in an inflammatory response. There is no consensus about the treatment of CAA, and the decision is taken case-by-case. More experience is needed to tackle these cases and long-term follow-up of patients with CCA development after DES is recommended.
